# A synthesis of major environmental-body size clines of the sexes within arthropod species

**DOI:** 10.1007/s00442-019-04428-7

**Published:** 2019-06-03

**Authors:** Curtis R. Horne, Andrew G. Hirst, David Atkinson

**Affiliations:** 1 School of Environmental Sciences, University of Liverpool, Liverpool, L69 3GP UK; 2 Centre for Ocean Life, National Institute for Aquatic Resources, Technical University of Denmark, Kemitorvet, 2800 Kgs. Lyngby, Denmark; 3 Institute of Integrative Biology, University of Liverpool, Liverpool, L69 7ZB UK

**Keywords:** Sexual size dimorphism, Biogeography, Temperature, Seasonality, Altitude

## Abstract

Body size at maturity often varies with environmental conditions, as well as between males and females within a species [termed Sexual Size Dimorphism (SSD)]. Variation in body size clines between the sexes can determine the degree to which SSD varies across environmental gradients. We use a meta-analytic approach to investigate whether major biogeographical and temporal (intra-annually across seasons) body size clines differ systematically between the sexes in arthropods. We consider 329 intra-specific environmental gradients in adult body size across latitude, altitude and with seasonal temperature variation, representing 126 arthropod species from 16 taxonomic orders. On average, we observe greater variability in male than female body size across latitude, consistent with the hypothesis that, over evolutionary time, directional selection has acted more strongly on male than female size. In contrast, neither sex exhibits consistently greater proportional changes in body size than the other sex across altitudinal or seasonal gradients, akin to earlier findings for plastic temperature-size responses measured in the laboratory. Variation in the degree to which body size gradients differ between the sexes cannot be explained by a range of potentially influential factors, including environment type (aquatic vs. terrestrial), voltinism, mean species’ body size, degree of SSD, or gradient direction. Ultimately, if we are to make better sense of the patterns (or lack thereof) in SSD across environmental gradients, we require a more detailed understanding of the underlying selective pressures driving clines in body size. Such understanding will provide a more comprehensive hypothesis-driven approach to explaining biogeographical and temporal variation in SSD.

**Electronic supplementary material:**

The online version of this article (10.1007/s00442-019-04428-7) contains supplementary material, which is available to authorized users.

## Introduction

Sexual size dimorphism (SSD) characterises the degree to which males and females differ in size within a species. Body size differences between the sexes have been related to dimorphic behavioural and ecological characteristics. For example, males are commonly larger than conspecific females in many endothermic vertebrates, especially those in which males compete with each other and hold territory or resources (e.g., Owens and Hartley 1998; Soulsbury et al. 2014). In contrast, in many ectothermic invertebrate species, including arthropods, the female is often the larger sex (e.g., Fairbairn 1997; Blanckenhorn et al. 2007a; Teder 2014). The larger body size of females in comparison to conspecific males has been attributed to their greater energy investment in the production and care of offspring, and the positive correlation between body size and fecundity (Slatkin 1984; Hedrick and Temeles 1989). Males invest relatively less energy in the production of gametes and often less in the care of offspring too; thus, males being larger may not result in an increased ability to produce more or fitter offspring. However, males maturing at a smaller size as a result of more rapid development could have a distinct advantage when the juvenile period is associated with high mortality rates, as may occur when males undertake risky mate-searching behaviour (Vollrath and Parker 1992; Savalli and Fox 1998; Blanckenhorn 2000; Kiørboe and Hirst 2008). Earlier maturation in males also means they are ready to mate with sexually maturing females—opportunities that later maturing males may miss (Wiklund and Fagerström 1977).

Variation in size at maturity within a species is affected by a range of environmental conditions. Such size variation can result from phenotypic plasticity, but also includes variation across populations, as observed across latitudinal gradients. Several biogeographic and biological ‘rules’ have consequently been proposed to describe systematic variation in body size. These include size clines over latitude, altitude, and with temperature and resource availability (Bergmann 1847; Atkinson 1994; Partridge and Coyne 1997; Blanckenhorn and Demont 2004; Chown and Gaston 2010; Forster et al. 2012; Shelomi 2012; Horne et al. 2015, 2017, 2018). The extent to which these body size clines differ between the sexes will determine the degree to which SSD varies across environmental gradients. Yet, very few studies have investigated sex-based variation in intra-specific adult body size clines, particularly across biogeographical and seasonal gradients. Latitudinal clines in body size have previously been compared between males and females in vertebrates and invertebrates, although the different metrics used to quantify variation in SSD resulted in contrasting outcomes. Males were the more variable sex when the ratios of sex-specific latitudinal slopes were compared (i.e., the relative difference between male and female latitudinal body size gradients), but neither sex was more variable when reduced major axis (RMA) slopes of male size on female size were used (Blanckenhorn et al. 2006). Variation in SSD across latitudinal-size (L-S) gradients, altitudinal-size (A-S) gradients, and with intra-annual temperature variation in the field, therefore, requires further investigation. Such analyses are necessary if we are to better understand sex-based differences in responses to the environment, as well as the likely reasons for changes in size at maturity. The need to understand environmental effects on size at maturity and SSD has been highlighted in a recent debate on the extent to which constraints on growth vs. the allometric scaling of costly reproductive output drives mature size and SSD (Barneche et al. 2018; Marshall and White 2018; Kearney 2019; Marshall and White 2019; Pauly 2019).

This study focuses on species of arthropod. Arthropoda is the most species-diverse phylum, which often dominates metazoan communities numerically in both aquatic (e.g., crustaceans) and terrestrial systems (e.g., insects) (Zhang 2013). Consequently, they form key food web components and can play an important role in the biogeochemical transformation of ecosystem materials (Turner 2004; Losey and Vaughan 2006). Changes in size at maturity in the field observed across latitude, altitude, and with seasonal temperature change (in this last case considering only multivoltine species), were recently synthesized for arthropod species (Horne et al. 2015, 2017, 2018). These studies revealed similarities in both the direction and magnitude of some of these major body size gradients, as well as consistency in the responses of certain taxa and between aquatic and terrestrial habitats (Forster et al. 2012; Horne et al. 2015, 2017). However, a detailed exploration of how these clines differed between males and females was not undertaken. The present study provides an opportunity to test the degree to which body size responses vary between the sexes across each of these major environmental gradients.

Effects of resource availability, juvenile density, and rearing temperature on variation in SSD in arthropods have previously been examined in short-term laboratory experiments (Teder and Tammaru 2005; Stillwell et al. 2010; Hirst et al. 2015; Rohner et al. 2018). However, where sex differences in body size plasticity have been observed, the underlying mechanisms and selective pressures are poorly understood. Changes in juvenile density and food quantity or quality have produced greater female size plasticity within arthropod species (Stillwell et al. 2010), many of which exhibit female-biased SSD, and thus the relative contribution of sex vs. body size to the degree of size plasticity is difficult to distinguish. A more recent study, which investigated sex-specific body size plasticity under laboratory conditions in holometabolous insects, found that the larger sex generally exhibited greater plasticity in response to environmental factors (including food quantity and temperature), indicating that selection on size, rather than on reproductive role, may be an important driver of sex-specific plasticity in insects (Rohner et al. 2018). These outcomes suggest that the energetic restrictions affecting body size plasticity may be acting to a greater extent on larger bodies. In contrast, a meta-analysis that included both aquatic and terrestrial arthropods found that laboratory temperature-size (T-S) responses did not vary systematically between the sexes (Hirst et al. 2015). These different outcomes suggest that there is generally a sex-dependent effect of food resources, but not temperature, on body size. Given the large number of environmental parameters that can vary in the field (including both resource availability and temperature), it is difficult to predict whether the degree of SSD will vary systematically across biogeographical and temporal gradients. Thus, in the present study we aim to establish whether:

1. Females exhibit the greatest proportional changes in body size across latitude, altitude, and with seasonal warming.
2. The larger sex exhibits the greatest proportional changes in body size across latitude, altitude, and with seasonal warming.
3. Neither of the sexes exhibits consistently greater proportional changes in body size than the other sex across these major environmental gradients.

We also investigate the degree to which any differences in these body size gradients between males and females within species depends on taxonomic and ecological attributes, including environment type (aquatic vs. terrestrial), voltinism, mean species body size, degree of SSD, and the direction of the size gradient.

## Methods

### Data collection

**Fig. 1:**
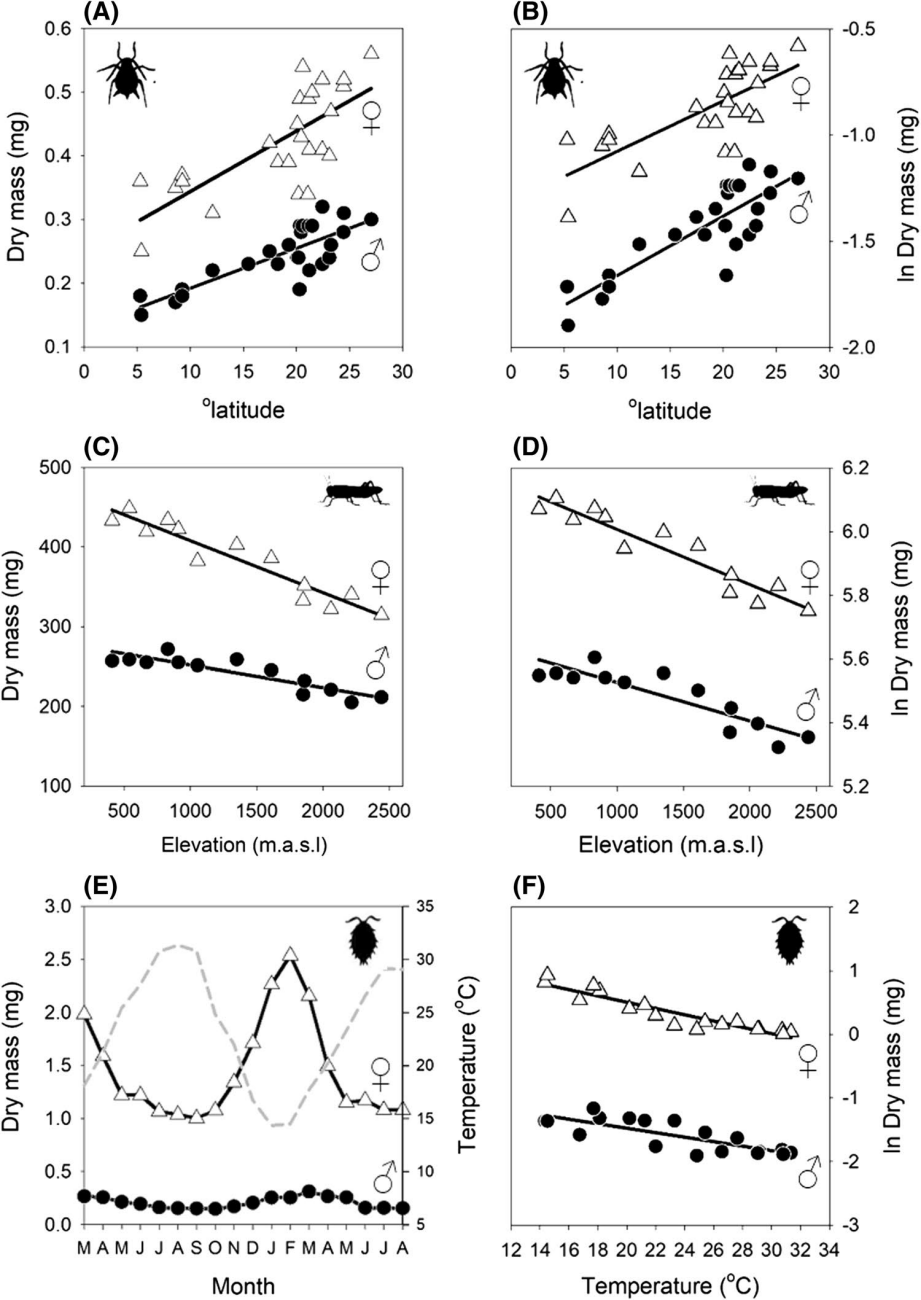
Examples of male (closed circles) and female (open triangles) body size clines across latitude (**a**, **b**), altitude (**c**, **d**) and with seasonal temperature variation (**e**, **f**). Left-hand panels show absolute changes in dry mass (mg), whilst right-hand panels show changes in natural log (ln) of dry mass, and thus relative change in body size. L-S data (**a**, **b**) is for *Dalbulus maidis* (Hemiptera), adapted from de Oliveira et al. (2004); A-S data (**c**, **d**) is for *Omocestus viridulus* (Orthoptera), adapted from Berner and Blanckenhorn (2006); seasonal temperature-size data (**e**, **f**) is for *Paracerceis sculpta* (aquatic Isopoda), adapted from Shuster and Guthrie (1999). Dashed grey line indicates seasonal variation in temperature in panel **e**. Note that males of *Paracerceis sculpta* coexist as three genetically distinct adult morphs; in panels **e** and **f** we show data for *y*-males, which mature most rapidly and are the smallest morph, resulting in particularly strong sexual size dimorphism. Despite the high degree of SSD, females and *y*-males exhibit very similar proportional changes in body size with seasonal warming (panel F). This highlights the importance of using an exponential equation form to compare body size clines, which avoids the scaling effects associated with using a linear regression, particularly in species with a high degree of SSD

The data compilations of Horne et al. (2015, 2017, 2018) provide data on size at maturity responses to latitude, altitude and seasonal temperature change in a wide range of arthropod species, including marine, freshwater and terrestrial-living forms. Of these, we used only adult size measurements from studies where size responses for males and females had been reported separately. We were careful to ensure that we only included measurements when data for both sexes had been collected following the same protocol, and across the same study transect or time period. Body size measurements were for field-collected individuals only, and thus common garden studies were excluded. Adult sizes in these data sets have been quantified using a variety of metrics (lengths, volumes, and different mass types). These measurements were converted to dry mass (mg) using intra-specific regressions. Where these were not available, we used regressions for closely related species, and occasionally more general inter-specific regressions. Our final data set consisted of 56 latitudinal-size clines representing 27 species, 129 altitudinal-size clines representing 50 species, and 144 seasonal temperature-size clines representing 52 species, examples of which are presented in Fig. 1. All data and conversions are detailed in Data Set S1 in the Supporting Information.

To quantify changes in body size, the OLS slopes of log_e_ dry mass vs. latitude (°), altitude (metres above sea level) and seasonal temperature (°C) were used to examine clines in body size for single species, separated by sex. This exponential equation form has the advantage of being a better fit than alternate transformations (linear, quadratic, and allometric), as judged by Akaike weights (Horne et al. 2015, 2017). In addition to fitting the empirical data well, this mathematical formulation is advantageous because it allows for an examination of relative size change and is unbiased by differences in absolute body size (also see Fig. 1). To provide a measure of relative size change for each species and sex along each environmental gradient (latitude, season, altitude), we transformed the OLS slopes into percentage change in dry mass per ^o^latitude, per  °C of seasonal temperature change, and per 150 m of elevation (approximating to a 1 °C change (Anslow and Shawn 2002)), respectively. The formula used was (exp^(slope)^ − 1) × 100 = % change in mass per unit (Forster et al. 2012). A negative percentage change indicates a decrease in size and a positive percentage change an increase. This allowed us to determine the relative difference in body size gradients between conspecific males and females (within single studies). Specifically, we used the degree of difference between male and female body size clines (% change in mass per unit) to calculate a size cline ratio, such that:

1 $${\text{Size cline ratio}} = \left( {{\text{larger size cline}}/{\text{smaller size cline}}} \right) - 1.$$

This approach returns symmetrical results around zero, regardless of which sex has the greater response. We assigned this ratio a positive value when males had the greater response and a negative value when the female response was greater. Given that we calculated body size clines using an exponential equation form, this metric provides a comparison of proportional body size change in males and females. This avoids the possible scaling effects encountered when using a linear regression, particularly in species with a high degree of SSD. For example, where both sexes exhibit the same proportional change in body size across environmental conditions, the slope of absolute size change would be greater in the larger sex. Were we to use a linear rather than exponential equation form, this would result in a size cline ratio that differs from zero, despite no change in SSD.

Note that the size cline ratio is derived from separate body size clines for males and females, and thus does not rely upon the body size of both sexes being measured at the exact same spatial or temporal point within a study (i.e., matched male–female values). An alternative size-scaling (allometric) approach, in which the log_10_ body size of one sex is plotted against that of the other (with the slope of an RMA regression then being derived), relies entirely on paired male and female body size data, which is not always obtained in ecological field studies. For this reason, we use the size cline ratio as the dependent variable in our analyses, as we believe this to be a more complete representation of SSD patterns. Indeed, using the allometric approach reduced the amount of data available in comparison to the size cline ratio method by ~ 60%. We repeated our analyses using an allometric approach, and summarise these findings, which largely support our conclusions, in the Supporting Information. We also utilise the allometric method in Table 1 to make direct comparisons with other published studies that have used this approach.

**Table 1:** Comparison of sex-specific plasticity in body mass in relation to environmental variables. Modified from Stillwell et al. (2010), with additions from Blanckenhorn et al. (2006) (which includes common garden experimental data), Hirst et al. (2015), and this study

Environmental variable (taxonomic group)	Which sex is more plastic?			Average degree of plasticity (CV among environments)			Source
	Females (no. studies with RMA slope < 1)	Males (no. studies with RMA slope > 1)	*X* ^2^	Female (%)	Male (%)	*T*	
Field-based clines:							
Latitude (Arthropoda)	8 (32.0%)	17 (68.0%)	2.56	15.3	17.2	− 1.81	This study
Altitude (Arthropoda but primarily Insecta)	32 (57.1%)	24 (42.8%)	0.88	12.0	11.5	0.58	This study
Seasonal temperature							
(Arthropoda but primarily Crustacea)	40 (60.6%)	26 (39.4%)	2.56	21.9	19.7	2.67**	This study
Latitude (Arthropoda)	17 (44.7%)	21 (55.3%)	0.24	5.50	5.54	0.27	Blanckenhorn et al. (2006)
Controlled laboratory-based clines:							
Temperature (Arthropoda)	55 (47.4%)	61 (52.6%)	0.22	12.3	12.1	0.41	Hirst et al. (2015)
Temperature (Insecta)	46 (48.9%)	48 (51.1%)	0.01	11.6	11.0	1.14	Hirst et al. (2015)
Larval density/larval competition							
Diet quantity (Insecta)	18 (72.0%)	7 (28.0%)	4.84*	16.0	12.2	3.42**	Stillwell et al. (2010)
Pathogenic infection (Insecta)	3 (50.0%)	3 (50.0%)	0.00	6.9	7.2	0.34	Stillwell et al. (2010)
Photoperiod (Insecta)	1 (16.7%)	5 (83.3%)	2.67	8.6	10.7	2.18	Stillwell et al. (2010)
Diet quality (Insecta)	83 (61.9%)	51 (39.1%)	7.64**	12.5	11.5	2.47*	Stillwell et al. (2010)

We followed the methodology of Stillwell et al. (2010), such that log_10_ male size is plotted on the *y*-axis, and log_10_ female size on the *x*-axis. Hence when the RMA slope is < 1 females are the more size responsive sex, and when the RMA slope is > 1 males are more size responsive. CV is the coefficient of variation of body size across the data within each study. Asterisks denote a significant difference between the sexes, where *p < 0.05, and **p < 0.01

In addition to the size cline ratio, we also used mean species body mass at the mid-latitude, mid-altitude or mid-temperature to calculate the absolute degree of SSD for each species within single studies, using the Sexual Dimorphism Index (SDI) of Lovich and Gibbons (1992), where:

2 $${\text{SDI}} = \left( {{\text{mass of larger sex}}/{\text{mass of smaller sex}}} \right) - 1.$$

We assigned this metric a positive value when males were the larger sex, and a negative value when females were larger, thus providing a measure of the relative difference in size between the sexes that varied symmetrically around zero. This allowed us to incorporate SDI as an independent variable in subsequent statistical analyses.

### Statistical analyses

All statistical analyses were conducted in R (version 3.4.1) (R Core Team 2014). For each of the three major body size gradients, we compared several candidate models to best predict within-species variation in the size cline ratio. Using the size cline ratio as the dependent variable, we began by incorporating different taxonomic and ecological traits as fixed variables in a global linear mixed effects model, created using the ‘lmer’ function in package lme4 (Bates et al. 2014). These included environment type (aquatic vs. terrestrial), voltinism (qualitative: one generation or less vs. multiple generations per year), mean species body size (calculated for females at the mid-latitude, mid-altitude and mid-temperature of each study), the direction of the size gradient (negative or positive), and SDI (calculated in Eq. 2). Note that voltinism was excluded when assessing seasonal temperature-size clines, as these comprised of multivoltine species only. Species are related and, therefore, not statistically independent, and our data set also included multiple size cline ratios for the same species; thus, we incorporated levels of taxonomic classification (class, order, family, and species) as nested (hierarchical) random effects on the intercept to help control for phylogeny (Koricheva et al. 2013). Furthermore, given that the size cline ratio was derived from data that varied in their goodness of fit between studies and species, we weighted this metric based on information quality (Koricheva et al. 2013). Specifically, size cline ratios were weighted by the inverse of the variance of the size cline slopes from which they were calculated. We recognise that our data set was derived from studies that adopted a population approach, in which the body size reported at a particular temperature, latitude or altitude is representative of a population mean rather than that of a single individual. Unfortunately, inconsistency between studies in the resolution of available data made it difficult to account for variation in information quality associated with each population mean. Nevertheless, it is noteworthy that we only included size clines from single studies, rather than combining size data from multiple studies that may vary greatly in their sampling protocol. Thus, within a cline, the number of individuals measured at each temperature, latitude or altitude should be reasonably consistent.

To examine which of our fixed variables best explained variation in the size cline ratio, we generated a set of candidate models from all the possible combinations of the global model terms using the ‘dredge’ function in the ‘MuMIn’ package (Barton 2017). Included in this candidate set was a null model, which contained no independent variables and predicted that the best estimate of the size cline ratio was the intercept only. We compared the complete list of models using Akaike’s Information Criterion (AIC), and the best model was identified as that with the lowest small-samples corrected AIC (AICc) (Burnham and Anderson 2002). Using package ‘AICcmodavg’ (Mazerolle 2014), we averaged over the whole set of candidate models (i.e., global model and all possible simpler models) to calculate the ‘full’ model-averaged coefficients for each of our fixed variables and determine their significance (*z*-statistic, *p* < 0.05). The ‘full’ average makes the assumption that each variable is included in every candidate model, but in some models the corresponding coefficient (and its respective variance) is set to zero. This reduces the tendency of biasing the estimated coefficients away from zero. For each of the three major body size gradients, we used the intercept from the null model (i.e., constant mean model) to infer an overall weighted-mean size cline ratio, which accounted for the non-independence between species, as well as variation in information quality of the data. Finally, for each environmental cline we used an *F* test to determine whether the size cline ratio differed significantly between taxonomic orders.

## Results

### Latitudinal-size clines

**Fig. 2:**
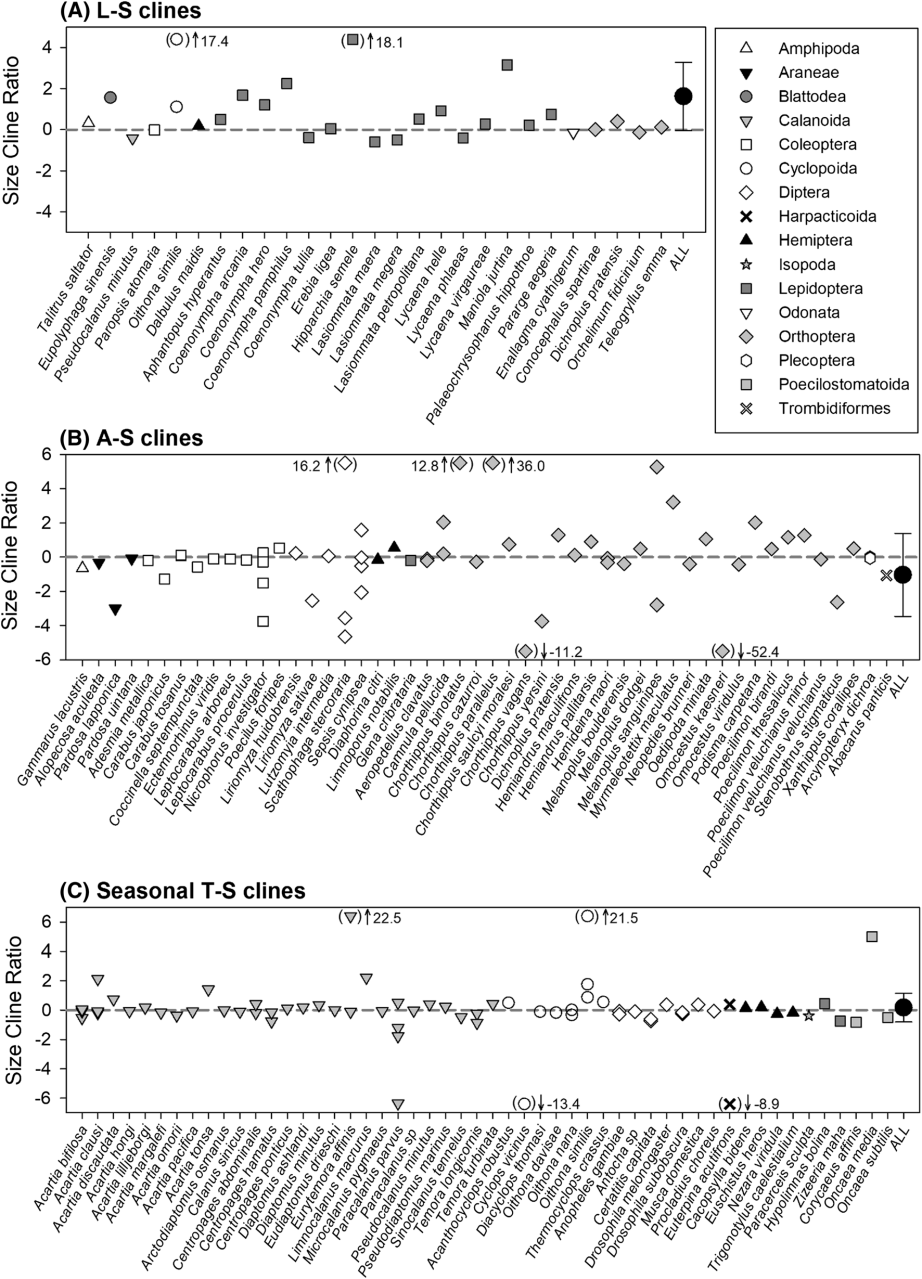
Size cline ratio*s* for **a** latitudinal-size (L-S) clines (*n* = 28), **b** altitudinal-size (A-S) clines (*n* = 64) and **c** seasonal temperature-size (T-S) clines (*n* = 72) for the arthropod species included in this study, categorized by taxonomic order. The horizontal dashed line denotes zero, i.e., no difference between male and female body size responses. Values greater than zero indicate more responsive male mass. Values less than zero indicate more responsive female mass. The overall weighted-mean size cline ratio (± 95% CI) is also shown for each environmental cline

Males exhibited stronger latitudinal-size clines relative to their conspecific females in 71% of cases. However, the overall weighted-mean size cline ratio (1.62 ± 1.66 95% CI), which accounted for the non-independence between species and variation in information quality, did not differ significantly from zero (*t*_5,23_ = 1.95, *p* = 0.06; Fig. 2a). Consequently, neither of the sexes exhibited consistently greater proportional changes in body size than the other sex across latitude. The best-supported model for explaining variation in the size cline ratio was a null model, which contained no independent variables and predicted that the best estimate of the size cline ratio was the intercept (see Table S1 in Supporting Information). After model averaging, none of the fixed variables included in our global model could significantly explain variation in the size cline ratio (see Table S7 for a summary of these outcomes). Neither did the size cline ratio vary significantly between taxonomic orders (*F*_8,19_ = 0.82, *p* = 0.59). Note than when using the alternative allometric approach, on average males exhibited significantly greater proportional changes in body size than females across latitude. However, as with the size cline ratio, none of the fixed variables included in our global model could significantly explain variation in the allometric slope between species (see Supporting Information).

### Altitudinal-size clines

Females exhibited stronger altitudinal-size clines relative to their conspecific males in 56% of cases. The overall weighted-mean size cline ratio (− 0.96 ± 2.22 95% CI) did not differ significantly from zero (*t*_5,50_ = − 0.86, *p* = 0.39; Fig. 2b). Thus, neither of the sexes exhibited consistently greater proportional changes in body size than the other sex across altitude. The best-supported model for explaining variation in the size cline ratio was a null model, which contained no independent variables and predicted that the best estimate of the dependent variable was the intercept (see Table S3). After model averaging, none of the fixed variables included in our global model could significantly explain variation in the size cline ratio (Table S7). Neither did the size cline ratio vary significantly between taxonomic orders (*F*_8,46_ = 0.11, *p* = 0.99). These outcomes are also corroborated by analysis using the alternative allometric approach (see Supporting Information).

### Seasonal temperature-size clines

Females exhibited stronger seasonal temperature-size clines relative to their conspecific males in 61% of cases. The overall weighted-mean size cline ratio (0.17 ± 0.97 95% CI) was not significantly different from zero (*t*_5,66_ = 0.34, *p* = 0.73; Fig. 2c). Thus, neither of the sexes exhibited consistently greater proportional changes in body size than the other sex with seasonal warming. The best-supported model for explaining variation in the size cline ratio was a null model, which contained no independent variables and predicted that the best estimate of the dependent variable was the intercept (see Table S5). After model averaging, none of the fixed variables included in our global model could significantly explain variation in the size cline ratio (Table S7). There was no significant difference in the size cline ratio between taxonomic orders (*F*_7,63_ = 0.44, *p* = 0.87). These outcomes were corroborated by analyses using the alternative allometric approach (see Supporting Information).

### Additional observations and considerations

For each of the environmental-body size clines, there were some particularly strong size cline ratios. Given that the body size cline of the less variable sex can be zero (i.e., the denominator in Eq. 1), theoretically the size cline ratio can be infinite. Thus, a very low denominator value compared with the numerator can generate very large ratios. Therefore, we also calculated the overall weighted-mean size cline ratio for each environmental cline when these strong outliers were excluded. Specifically, we excluded size cline ratios that ranged above and below 1.5 × the interquartile range. This resulted in the removal of 3, 14 and 11 outliers from latitudinal-, altitudinal-, and seasonal temperature-size clines, respectively. As before, the mean size cline ratio for both altitudinal-size clines (0.10 ± 0.33 95% CI) and seasonal temperature-size clines (− 0.09 ± 0.10 95% CI) did not differ significantly from zero (*t*_6,35_ = 0.57, *p* = 0.59 and *t*_6,54_ = − 1.74, *p* = 0.09, respectively). When these outliers for latitudinal-size clines were excluded, the mean size cline ratio became significantly positive (0.38 ± 0.29 95% CI; *t*_5,20_ = 2.66, *p* = 0.01), suggesting greater variation in male than female body size with latitude.

Across all three major body size gradients, there were a small number of cases (*n* = 18) where the direction of the size gradients differed between males and females within a species (i.e., whereas one sex increased in size, the other decreased in size). Yet in each case, the slope of at least one of these paired size gradients, and in most cases both (*n* = 14), did not differ significantly from zero (determined by the 95% CIs overlapping with zero). Thus, we find strong and consistent evidence that within a species, males and females share the same sign (positive or negative) in the environmental-body size clines we have tested.

## Discussion

To our knowledge, this study provides the largest quantitative comparison of male and female biogeographical and temporal (seasonal) body size gradients to date in arthropods, including marine, freshwater and terrestrial species. Given the contrasting outcomes from recent studies investigating sex-specific body size plasticity under laboratory conditions (Table 1), we combined body size data from multiple species and studies to provide a field-based comparison to these earlier findings.

Blanckenhorn et al. (2006) previously compared latitudinal-size clines between males and females in vertebrates and invertebrates, finding that the different metrics used to quantify variation in SSD led to contrasting outcomes. Males were the more variable sex when the ratios of sex-specific latitudinal slopes (i.e., size cline ratio) were compared, but neither sex was more variable when an allometric approach was used (Blanckenhorn et al. 2006). In our assessment of latitudinal-size clines, males exhibited greater L-S clines than females in over two-thirds of our data set, and after removing particularly strong outliers, the weighted-mean size cline ratio was significantly greater than zero, indicating greater variability in male than female body size across latitude. Moreover, this same pattern was evident following analysis using the allometric approach (see Supporting Information). However, we note that this allometric approach (which relies upon paired male and female values) reduced the amount of latitudinal-body size data available by almost two-thirds.

Of the three environmental gradient types examined, latitudinal-size clines are the most likely to include not just phenotypically plastic effects, but also genetic differences between populations. Evidence of greater variability in male than female size against latitude is consistent with the hypothesis that, over evolutionary time, directional selection has acted more strongly on male than female size (Fairbairn 1997). This hypothesis may be developed further, given that a large proportion of our latitudinal-size clines were for Lepidoptera, many of which exhibit protandry (i.e., earlier male emergence) and show converse latitudinal-size clines, decreasing in size towards the poles. This finding, therefore, supports the suggestion that, due to seasonal time constraints at higher latitudes, particularly strong selection for earlier male emergence (and thus smaller size) may be driving greater variability in male than female body size across latitudinal gradients, providing a possible explanation for the observed patterns (Roff 1980; Blanckenhorn et al. 2007b).

In contrast to latitudinal gradients, altitudinal-size clines and seasonal temperature-size clines are somewhat less likely to be influenced by genetic differences between populations and more so by phenotypic plasticity. Indeed, we find that neither of the sexes exhibit consistently greater proportional changes in body size than the other sex across altitudinal and seasonal gradients, akin to earlier findings reported for plastic temperature-size responses measured in the laboratory (Hirst et al. 2015). Although changes in juvenile density and food quantity/quality have been shown to produce greater female size plasticity within arthropod species (Stillwell et al. 2010), the environmental gradients we examine here are strongly characterized by predictable variation in temperature, whereas gradients in other variables such as food quality and juvenile density are relatively less predictable. Furthermore, whereas Rohner et al. (2018) found that the larger sex generally exhibited greater plasticity in response to environmental factors in insects (including food quantity and temperature), variation in the size cline ratio in our study could not be explained by any combination of taxonomic and ecological traits, including the magnitude and direction of SSD. Therefore, we find no evidence to suggest that body size plasticity is generally greater in the larger sex.

A tentative explanation for the lack of systematic differences between male and female altitudinal and seasonal body size gradients may lie in their ontogenetic establishment, particularly if these environmental clines are primarily the result of body size plasticity in response to developmental temperature. A meta-analysis investigating the proximate cause of sexual size dimorphism in insects concluded that in many species (79%), the larger sex also had a longer larval development time (Teder 2014). Furthermore, greater differences in larval development time between the sexes corresponded with a greater degree of SSD in a diverse range of insect clades (Teder 2014). These findings suggest that prolonged development time in the larger sex plays an important role in establishing SSD, although differences in the growth rate of males and females has also been proposed as the primary mechanism (Blanckenhorn et al. 2007a). We may predict that the later developing sex would exhibit stronger body size clines if we make two assumptions. First, SSD arises primarily from longer development time in the larger sex, whether this be through prolonged development of several consecutive instars (Tammaru et al. 2010), or through the addition of an extra instar at the end of ontogeny (Esperk and Tammaru 2006). Second, temperature-size responses are established gradually over ontogeny, such that eggs show little or no response and the strength of the response accumulates over time (Forster et al. 2011). Furthermore, we would expect a stronger size cline ratio in those species with a higher degree of SSD. Yet, we do not observe such patterns. This mis-match between prediction and observation may arise because the second assumption appears not to hold, at least in those few arthropods studied (Forster and Hirst 2012; Horne et al. 2019). The temperature-size responses of these species show no consistent change (strengthening or weakening) during the second half of ontogeny (Forster and Hirst 2012; Horne et al. 2019). If the ontogenetically early onset of body size clines is widespread among arthropods, this may explain why both sexes show a similar degree of plasticity in adult size, even if the larger sex has a markedly longer development time. In contrast, the effects of other environmental variables such as food quality/quantity may continue to accumulate across the whole of ontogeny. Our speculative proposal for such differences requires further empirical examination and testing.

Although we find no systematic patterns in the size cline ratio across altitudinal and seasonal gradients, considerable variation exists in this metric between species (Fig. 2). Although it is difficult to conduct a detailed assessment of the life history, physiology and population dynamics of every species in our data set, we make two suggestions to improve understanding. First, rather than treating body size as an isolated trait, further studies should incorporate co-adaptation of responses to the environment (Angilletta et al. 2006). Specifically, differences in body size at maturity can arise from differences in growth, development rates (e.g., affecting protandry), or both, and all these traits will be selected according to their influences on and by the schedules of mortality and reproduction (e.g., fecundity potential) (Roff 1986; Marshall and White 2018). Thus, we advocate treating life-history differences between the sexes as a co-adapted whole and identifying specific environmental (including social) conditions that generate these differences. Second, particular case studies may help elucidate the patterns (or lack thereof) in SSD across environmental gradients. For example, considerable variation exists in the size cline ratio across altitudinal gradients within the Orthoptera. Of these, data for *Chorthippus cazurroi*, *C. parallelus* and *C. yersini* were derived from Laiolo et al. (2013), who investigated intra-specific variation in SSD in mountain grasshopper communities. *Chorthippus yersini* exhibits a particularly strong negative size cline ratio (i.e., greater variability in female size; Fig. 2b). As the authors point out, this may be explained by the fact that females of a phylogenetically similar species produce additional instars when raised at higher temperatures and with higher food quality (Hassall and Grayson 1987). Prolonged development through the addition of extra instars during ontogeny would allow females to become substantially larger than males in favourable conditions, and thus could provide a proximate explanation for the greater variation in female than male size observed across altitude in this species (Laiolo et al. 2013). In contrast, *C. parallelus* exhibits a very strong positive size cline ratio (i.e., greater variability in male size; Fig. 2b) and is one of the few species in the *Chorthippus* genus for which females cannot alter the number of instars during development (Schädler and Witsack 1999). This fixed instar number may act to constrain variability in female body size across altitudinal gradients; hence the observations of Laiolo et al. (2013). Other studies have also identified sex-biased plasticity in the physiological mechanisms controlling insect body size during ontogeny, including the hormonal pathways regulating growth rate and critical size (Davidowitz et al. 2004; Stillwell and Davidowitz 2010; Testa et al. 2013; Nijhout et al. 2014; Stillwell et al. 2014). However, the mechanism(s) leading to variation in male and female body size responses are unlikely to be universal, particularly as these plastic size responses are not just limited to arthropods (Atkinson 1994; Blanckenhorn et al. 2006; Forster et al. 2012).

The data presented here represents only a small fraction of all arthropod species, with some taxa better represented than others. Furthermore, variation in abiotic and biotic conditions across environmental gradients will undoubtedly vary between study locations, further confounding any potential patterns. Ultimately, if we are to make more broad-scale predictions about sex-based differences in response to the environment, we require a more detailed understanding of the underlying selective pressures driving clines in body size. Such understanding will provide a more comprehensive hypothesis-driven approach to explaining biogeographical and temporal variation in SSD.

## Electronic supplementary material

Below is the link to the electronic supplementary material.

Supplementary material 1 (XLSX 708 kb)

Supplementary material 2 (DOCX 496 kb)

## Data Availability

Raw data used in this study are available in Supporting Information Data Set S1.
