# Supplementary material for: A synthesis of major environmental-body size clines of the sexes within arthropod species
Source: Oecologia. 2019 Jun 3;190(2):343–53. doi: 10.1007/s00442-019-04428-7 (PMC6571078; doi:10.1007/s00442-019-04428-7)
Supplement: Supplementary file 2 — Supplementary material 2 (DOCX 496 kb) [file 442_2019_4428_MOESM2_ESM.docx]

**Supporting Information**

**An alternative allometric approach**

In the main text we used *Size Cline Ratios* to quantify the degree to which body size gradients differed between con-specific males and females (within single studies). Here we present the outcomes following an allometric approach, by which an RMA regression of the log_10_ body size of one sex against the log_10_ of the other sex is plotted (Fairbairn & Preziosi, 1994; Fairbairn, 1997).

In this method, each individual data point represents paired size measurements at different latitudes, altitudes or seasonal temperatures. The RMA slope provides a quantitative expression of how the sizes of the sexes change together. When plotting this regression, the more size-responsive sex was represented on the y-axis and the less size-responsive on the x-axis, such that the slope was ≥ 1. We subtracted 1 from the slope and set the values as positive when male size was the more responsive, and negative when female size was the more responsive. As above, this method ensures that results vary symmetrically around zero based on equivalent differences between the sexes, regardless of which sex shows the greater response. We term this value the *RMA_Index_*. A value of zero indicates equal covariation in the size of both of the sexes, whereas increasingly positive values indicate greater variation in male size, and increasingly negative values indicate greater variation in female size.

The *Size Cline Ratio* is generally more variable than the *RMA_Index_*, at times producing rather strong negative and positive values (see Figure S1 for comparisons of these metrics). This statistical effect arises because the size cline of the less variable sex can be zero or close to this value (i.e. the denominator in equation 2 of the main text), and thus the *Size Cline Ratio* can be driven to be a very large number. However, on average, the *Size Cline Ratio* and *RMA_Index_* produce somewhat similar patterns for each of the environmental gradient types.

To examine within-species variation in the degree of SSD for each of the three major body size gradients, we followed exactly the same methodology as described in the main text, simply replacing the S*ize Cline Ratio* with the *RMA_Index_* as the dependent variable in our models. Note that when weighting by information quality, *RMA_Index_* values were weighted by the inverse of the variance of the allometric slopes from which they were derived.

*Latitudinal-Size Clines*

The overall weighted-mean *RMA_Index_* (0.41±0.36 95% CI), which accounted for the non-independence between species and variation in information quality, did differ significantly from zero (*t*_23_=2.29, p=0.03). Thus, on average, males exhibited greater proportional changes in body size than females across latitude. The best supported model for explaining variation in the *RMA_Index_* was a null model, which contained no independent variables and predicted that the best estimate of the *RMA_Index_* was the intercept (Table S2). After model averaging, none of the fixed variables included in our global model could significantly explain variation in the *RMA_Index_* (see Table S7 for a summary of these outcomes). Neither did the *RMA_Index_* vary significantly between taxonomic orders (*F*_6,16_=0.33, p=0.91).

*Altitudinal-Size Clines*

The overall weighted-mean *RMA_Index_* (-0.55±0.84 95% CI) did not differ significantly from zero (*t*_6_=-1.31, p=0.24). Thus, neither of the sexes exhibited consistently greater proportional changes in body size than the other across altitude. The best supported model for explaining variation in the *RMA_Index_* contained mean species body size as a single fixed parameter (Table S4). However, after model averaging, none of the fixed variables included in the global model could significantly explain variation in the *RMA_Index_* (Table S7). Neither did the *RMA_Index_* vary significantly between taxonomic orders (*F*_8,40_=1.74, p=0.12).

*Seasonal Temperature-Size Clines*

The overall weighted-mean *RMA_Index_* (-0.08±0.14 95% CI) was not significantly different from zero (*t*_47_=-1.18, p=0.25). Thus, neither of the sexes exhibited consistently greater proportional changes in body size than the other with seasonal warming. The best supported model for explaining variation in the *RMA_Index_* was a null model, which contained no independent variables and predicted that the best estimate of the dependent variable was the intercept (Table S6). After model averaging, none of the fixed variables included in our global model could significantly explain variation in the *RMA_Index_* (Table S7). There was no significant difference in the *RMA_Index_* between taxonomic orders (*F*_7,55_=1.49, p=0.19).


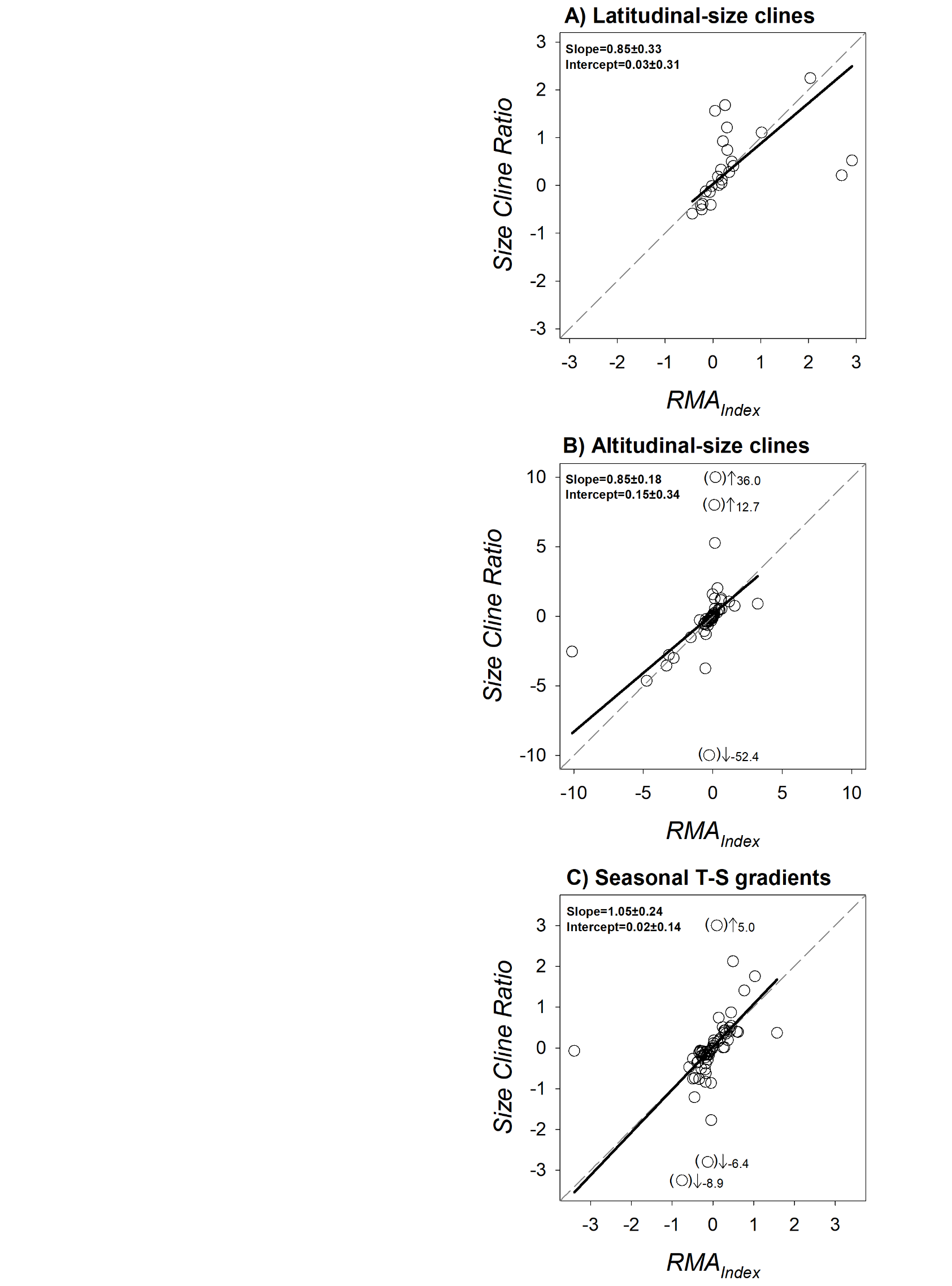

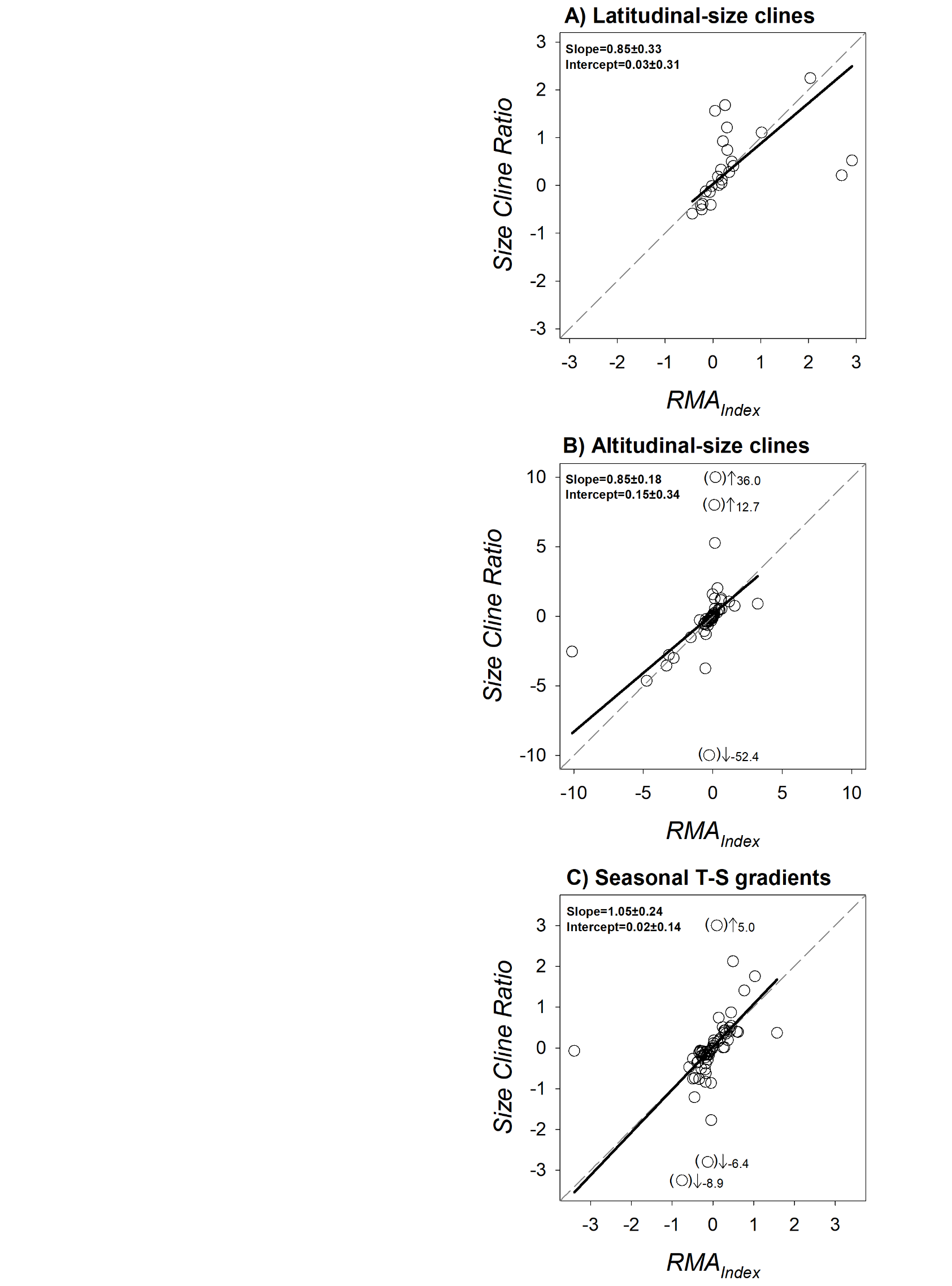

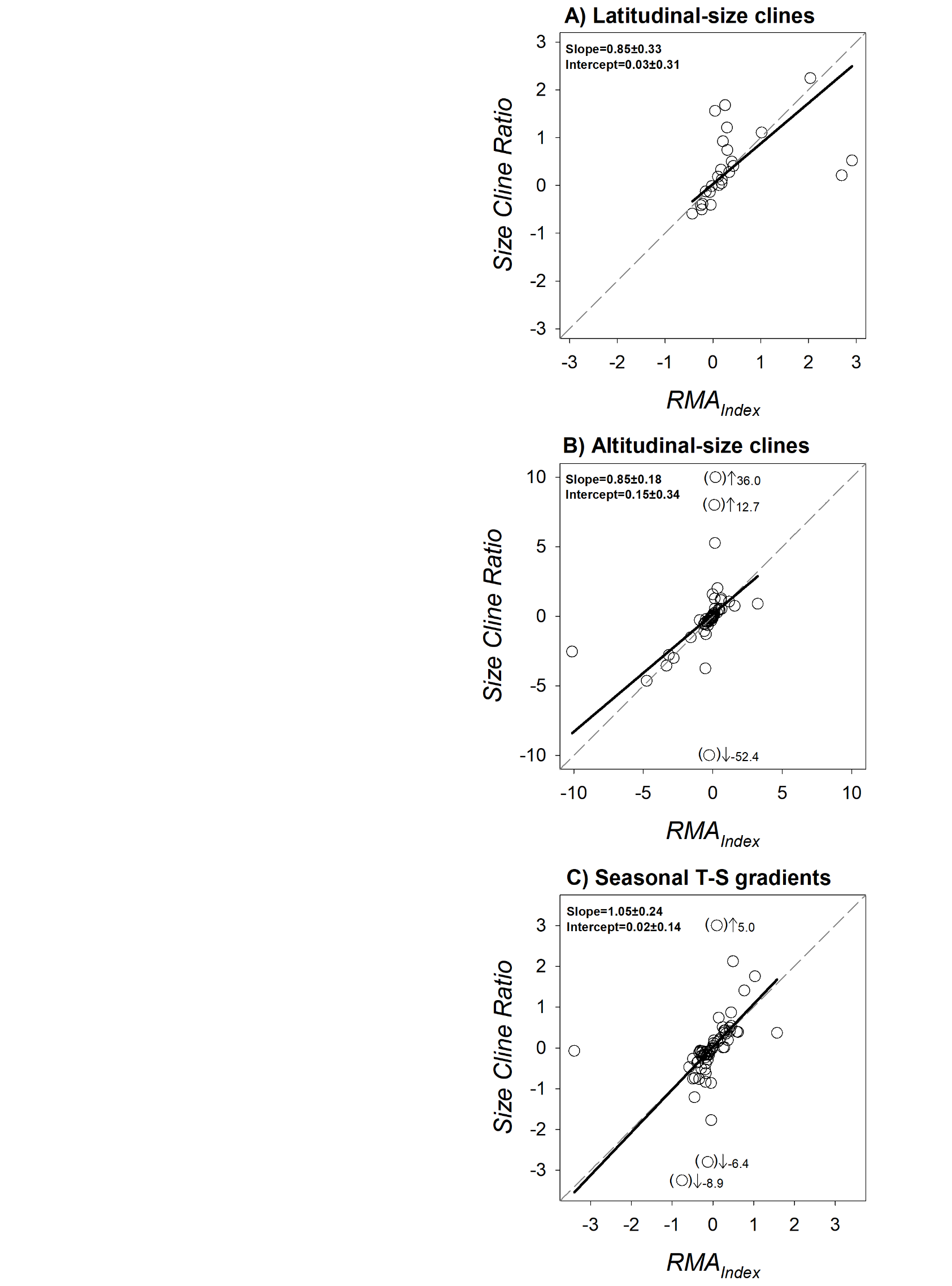
**Figure S1.** Comparison of the outcomes from the *Size Cline Ratio* method versus the *RMA_Index_* method, where both are measures of the difference in the responses of the sexes to environmental conditions. A. latitudinal-size clines, B. altitudinal-size clines, and C. seasonal temperature-size gradients. In each graph, the slope and intercept (± 95% CI) are given for the RMA regression through the data. Extreme outliers are highlighted in brackets, and were excluded when calculating the RMA regression. In all cases, the slope of the RMA regression does not differ significantly from 1, whilst the intercept does not differ significantly from zero (inferred from the 95% confidence intervals). For reference, the dashed line indicates a 1:1 relationship between the two metrics.

**Table S1.** AIC output comparing the relative strength of candidate models in explaining variation in the *Size Cline Ratio* for latitudinal-size clines.

Notes: The parameters included in each model are indicated with a plus sign (+).The number of parameters (K), AICc, and the difference between a model’s AICc and the lowest AICc (i.e. ΔAICc) is also shown. Akaike weights (*wi*) denote the probability of a given model being the best fit model in the candidate set. Mass is the mean female body mass at the mid-latitude of each study. In all models, levels of taxonomic classification (class, order, family, and species) were incorporated as nested (hierarchical) random effects on the intercept to help control for phylogeny. To account for variation in information quality between studies and species, *Size Cline Ratios* (i.e. the dependent variable) were also weighted by the inverse of the variance of the size cline slopes from which they were derived.

**Table S2.** AIC output comparing the relative strength of candidate models in explaining variation in the *RMA_Index_* for latitudinal-size clines, where *RMA_Index_* values were weighted by the inverse of the variance of the allometric slopes from which they were derived.

Notes: The parameters included in each model are indicated with a plus sign (+).The number of parameters (K), AICc, and the difference between a model’s AICc and the lowest AICc (i.e. ΔAICc) is also shown. Akaike weights (*wi*) denote the probability of a given model being the best fit model in the candidate set. Mass is the mean female body mass at the mid-latitude of each individual study. In all models, levels of taxonomic classification (class, order, family, and species) were incorporated as nested (hierarchical) random effects on the intercept to help control for phylogeny. To account for variation in information quality between studies and species, *RMA_Index_* values (i.e. the dependent variable) were also weighted by the inverse of the variance of the allometric slopes from which they were derived.

**Table S3.** AIC output comparing the relative strength of candidate models in explaining variation in the *Size Cline Ratio* for altitudinal-size clines.

Notes: The parameters included in each model are indicated with a plus sign (+).The number of parameters (K), AICc, and the difference between a model’s AICc and the lowest AICc (i.e. ΔAICc) is also shown. Akaike weights (*wi*) denote the probability of a given model being the best fit model in the candidate set. Mass is the mean female body mass at the mid-altitude of each individual study. In all models, levels of taxonomic classification (class, order, family, and species) were incorporated as nested (hierarchical) random effects on the intercept to help control for phylogeny. To account for variation in information quality between studies and species, *Size Cline Ratios* (i.e. the dependent variable) were also weighted by the inverse of the variance of the size cline slopes from which they were derived.

**Table S4.** AIC output comparing the relative strength of candidate models in explaining variation in the *RMA_Index_* for altitudinal-size clines, where *RMA_Index_* values were weighted by the inverse of the variance of the allometric slopes from which they were derived.

Notes: The parameters included in each model are indicated with a plus sign (+).The number of parameters (K), AICc, and the difference between a model’s AICc and the lowest AICc (i.e. ΔAICc) is also shown. Akaike weights (*wi*) denote the probability of a given model being the best fit model in the candidate set. Mass is the mean female body mass at the mid-altitude of each individual study. In all models, levels of taxonomic classification (class, order, family, and species) were incorporated as nested (hierarchical) random effects on the intercept to help control for phylogeny. To account for variation in information quality between studies and species, *RMA_Index_* values (i.e. the dependent variable) were also weighted by the inverse of the variance of the allometric slopes from which they were derived.

**Table S5.** AIC output comparing the relative strength of candidate models in explaining variation in the *Size Cline Ratio* for seasonal temperature-size gradients.

Notes: The parameters included in each model are indicated with a plus sign (+).The number of parameters (K), AICc, and the difference between a model’s AICc and the lowest AICc (i.e. ΔAICc) is also shown. Akaike weights (*wi*) denote the probability of a given model being the best fit model in the candidate set. Mass is the mean female body mass at the mid-temperature of each individual study. In all models, levels of taxonomic classification (class, order, family, and species) were incorporated as nested (hierarchical) random effects on the intercept to help control for phylogeny. To account for variation in information quality between studies and species, *Size Cline Ratios* (i.e. the dependent variable) were also weighted by the inverse of the variance of the size cline slopes from which they were derived.

**Table S6.** AIC output comparing the relative strength of candidate models in explaining variation in the *RMA_Index_* for seasonal temperature-size gradients, where *RMA_Index_* values were weighted by the inverse of the variance of the allometric slopes from which they were derived.

Notes: The parameters included in each model are indicated with a plus sign (+).The number of parameters (K), AICc, and the difference between a model’s AICc and the lowest AICc (i.e. ΔAICc) is also shown. Akaike weights (*wi*) denote the probability of a given model being the best fit model in the candidate set. Mass is the mean female body mass at the mid-temperature of each individual study. In all models, levels of taxonomic classification (class, order, family, and species) were incorporated as nested (hierarchical) random effects on the intercept to help control for phylogeny. To account for variation in information quality between studies and species, *RMA_Index_* values (i.e. the dependent variable) were also weighted by the inverse of the variance of the allometric slopes from which they were derived.

**Table S7.** ‘Full’ model-averaged coefficients for each of the fixed variables in our global linear mixed effects model, averaged over the whole set of candidate models (see Methods in main text). Outcomes are shown for each of the three major body size gradients (latitude-, altitude-, and seasonal temperature-size clines), both when using the *Size Cline Ratio* and *RMA_Index_* as the dependent variable. Confidence intervals, z-values and p-values are also shown. In all cases none of the fixed variables significantly explained variation in the *Size Cline Ratio* or *RMA_Index._*

**References**

Fairbairn, D.J. (1997) Allometry for sexual size dimorphism: Pattern and process in the coevolution of body size in males and females. *Annual Review of Ecology and Systematics*, **28**, 659-687.

Fairbairn, D.J. & Preziosi, R.F. (1994) Sexual selection and the evolution of allometry for sexual size dimorphism in the water strider, *Aquarius remigis*. *The American Naturalist*, **144**, 101-118.
